# Supplementary figures and images for: Assessment of corneal epithelial thickness mapping in epithelial basement membrane dystrophy
Source: PLoS One. 2020 Nov 25;15(11):e0239124. doi: 10.1371/journal.pone.0239124 (PMC7688121; doi:10.1371/journal.pone.0239124)

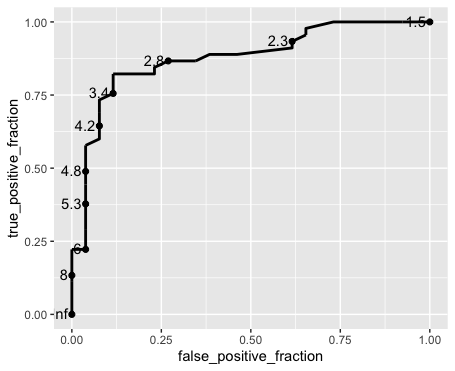

Supplement: S1 Fig — (TIFF) [file pone.0239124.s001.tiff]
