# Supplementary material for: Assessment of corneal epithelial thickness mapping in epithelial basement membrane dystrophy
Source: PLoS One. 2020 Nov 25;15(11):e0239124. doi: 10.1371/journal.pone.0239124 (PMC7688121; doi:10.1371/journal.pone.0239124)
Supplement: Attachment — Submitted filename: Response to ReviewersPO.docx [file pone.0239124.s002.docx]

**Response to Reviewers**

**Response to the academic editor**

| **Comment** | **Response** | **Changes made to the original version** |
| --- | --- | --- |
| The rationale for the study is not well defined, and this includes reviewing the Introduction, which is lengthy, and not clear in sections. For example, lines 67 to 111 could be significantly edited and the rationale for the study noted clearly at the end of this section, including the comparison with the dry eye group of patients | Thank you for you remark, we have shortened the introduction and clarified the rationale of the study. In particular we have deleted the part on the IVCM. | Line 98-198: “Fourier-domain OCT is an in vivo and non-contact technique that enables quick measurement of corneal epithelial thickness by obtaining high-resolution, cross-sectional imaging of biological tissues (11) and an accurate assessment of the corneal layers (12). With great reliability and repeatability, OCT has been chosen in several studies to evaluate the corneal epithelial thickness in ocular diseases (12,13). Given the implication of the epithelium in this corneal dystrophy, the study of the mapping of the corneal thickness seems to be interesting to characterize this pathology. However, to our knowledge no study has referred to the features of corneal epithelial thickness in EBMD. Therefore, we investigated the features of the corneal epithelial Fourier-domain OCT thickness map in EBMD patients compared to normal subjects and DED patients and we aimed to assess the diagnostic capacity of OCT for this pathology. We have chosen to compare the measures also with patients with dry eye for DED could be a confounding factor with epithelial irregularities and persistent or recurrent epithelial defects and we wished to evaluate whether those irregularities would match or not and if epithelial mapping could show different and/or specific patterns in either disease. Indeed, in DED tear film abnormalities are responsible for osmotic and inflammatory stresses, which combine with mechanical stress to promote epithelial abnormalities and defects, and prevent epithelium to recover properly (14,15).  Poor quality of vision, including glare, ghosting, blurriness, poor contrast, and haloes and starbursts can greatly decrease the quality of life of patients suffering from EBMD. However, to date no clear data exist showing a direct correlation between morphologic analyses with OCT or IVCM and objective parameters of optical quality in EBMD. The optical quality analysis system (OQAS; OQAS II; Visiometrics S.L., Tarrasa, Spain) is a noninvasive instrument that can be used to provide an objective evaluation of the optical quality of ocular structures. The OQAS is an useful tool to better evaluate visual function in patients with EBMD.  Thus, in this study, we aim to investigate the features of the corneal epithelial Fourier-domain OCT thickness map in EBMD patients and to assess the relationship between visual quality and OCT thickness mapping in EMBD compared to DED and normal patients.” |
| The full details of the study design are provided (see also Reviewers 1 and 2). Further, the details of the EBMD patients are not detailed. Were the grades of EBMD similar; were cysts present in any patients? Were there differences in the length of time since diagnosed? How was the EBMD being managed for each patient (e.g. bandage contact lens, regular debridement, laser etc). These will impact on epithelial basement membrane morphology and thickening overtime. | We have specified the criteria for inclusion of patients with EBMD. | Line 150-158: “. EBMD patients presenting symptomatic recurrent corneal erosions, irritation, or visual symptoms with morphological slit-lamp findings of EBMD were enrolled. Patients were considered to have recurrent erosions if they had painful episodes on awakening associated with typical slit-lamp findings of epithelial irregularity. Eyes were considered to have morphological slit-lamp findings of EBMD if fingerprint lines, microcysts, geographic maps, epithelial dots, were seen on slit-lamp examination (4,5). None of the EBMD patients had received bandage contact lens, or undergone epithelial debridement in the previous three months. Previous phototherapeutic keratectomy was an exclusion criteria.” |
| The issue of lid laxity or tightness for each patient requires comment, given a main conclusion relates to the action/force of the upper eyelid being involved in the increased central and inferior epithelial thickness. There were older patients in this group, where lid laxity is a common feature. Furthermore, differences in epithelial nerve density in different corneal regions could also be involved, as well the greater lid coverage of the upper cornea with relatively reduced oxygen levels. | Thank you for raising these very interesting points. We did not actually note the laxity of the eyelid which seems to play a role in the arrangement of the epithelium according to our study. The distribution of nerve density with its trophic role for the epithelium may also play a role. We added these points in the discussion. | Line 585-593: “Since we formulate the hypothesis of the involvement of the eyelid-epithelium interface to explain this pattern of epithelial thickening in this dystrophy, it would be interesting to study the impact of the lid laxity or tightness on the arrangement of the corneal epithelium. As well the greater lid coverage of the upper cornea which relatively reduced oxygen levels should be investigated (25). Furthermore, differences in epithelial nerve density in different corneal regions could also be involved and should be studied. Indeed, Bouheraoua et al. were able to show that decreased subbasal nerve plexus density led to a thinned corneal epithelium after 360-degre laser retinopexy (26).” |
| Details of the dry eye groups are also limited with no details of grade/type and duration. | Indeed, that's correct, we discuss this limit in the discussion part | Line 642-645: “, in this study all stages and causes of DED were included. Yet we know that the inflammatory process induces epithelial proliferation (38), whereas nerve damage results in thinning of the corneal epithelium (39).” |
| The Discussion requires editing - for example, the section from lines 282 to 298 related to slit lamp versus OCT and applications, does not really add to the critical assessment of the findings, and should be summarised or removed. | The have shortened the section related to IVCM versus OCT | See discussion |
| The final sentence refers to the OCT characterisation providing a structural explanation for the behaviour of the epithelium. Can the authors please explain what this means? As the study is cross-sectional and epithelial growth is truly dynamic, am unsure of this statement and its relevance. | We agree that this study do not give an explanation but rather an insight of the behavior of the epithelium in EBMD. Indeed this study, we demonstrated that the topography of the epithelium in EBMD presents a certain pattern with a thickening of the epithelium in the central and inferior regions. A hypothesis for this pattern could be formulated as the result of the interaction between the eyelid and the ocular surface especially in this dystrophy in which there is a lack of adhesion of epithelial cells to the basement membrane. | We have modified the final sentence to:  line 677-681 “The established characterization of this epithelial accumulation in the lower part of the cornea suggests an explanation of the behavior of the epithelium in this dystrophy and provides a simple and non-invasive tool to monitor patients with EBMD, capable of supporting future research and treatment strategies.” |

**Response to reviewer #1**

| Comment | Response | Changes made to the original version |
| --- | --- | --- |
| Line 135: were standardised questions used to assess symptoms related to either DED or EBMD? Were the conditions graded for each patient as this will have an impact on interpretation of results. | Even if it would have been interesting, we did not use a vision / life quality questionnaire and we did not grade the pathologies. These limits are discussed in the discussion section. | Line 608-610: “It would be interesting to study if, as we think, the OSI index is also correlated with the subjective quality of vision reported by patients with EBMD.”  Line 670-672: “Another limitation of the study is that we did not grade the severity of patients with EBMD or DED. It would be interesting to study if the irregularity of the epithelium is more important that the dystrophy is severe or that the functional complaints are important.” |
| Line 137: were patients undergoing treatment/ocular medications also excluded? | Patients under ocular medications weren’t excluded in the dry eye and EBMD groups |  |
| Line 150: are there been any studies investigating repeatability of this OCT on epithelial thickness measurement? | Two studies have investigated repeatability and reliability of this OCT on epithelial thickness measurement and found great reliability and repeatability.  - Sin S, Simpson TL. The Repeatability of Corneal and Corneal Epithelial Thickness Measurements Using Optical Coherence Tomography. *Optom Vis Sci.* 2006 Jun 1;83(6):360–5.  - Ma JX, Wang L, Weikert MP, Montes de Oca I, Koch DD. Evaluation of the Repeatability and Reproducibility of Corneal Epithelial Thickness Mapping for a 9-mm Zone Using Optical Coherence Tomography. *Cornea*. 2019 Jan;38(1):67–73. | Lines 103-105: “Fourier-domain OCT is a non-contact technique that enables measurement of corneal epithelial thickness (11) and an accurate assessment of the corneal layers (12) with great reliability and repeatability. To our knowledge no study has referred to the features of corneal epithelial thickness in EBMD.” |
| Line 157: please define each of the different sections within the cornea i.e. provide boundaries of the cornea is a representative of the superior cornea? How many measurements per eye were taken? Why select superior and inferior corneal locations only? | On the examination report provided by the manufacturer, the epithelial thickness values in the central 7 mm are presented: the upper and lower epithelial thickness in the area of 2 to 7 mm, the minimum and maximum thicknesses as well as the difference between these two thicknesses and the standard deviation of thickness in the evaluated area.  The measurements were repeated until a sufficient signal index was obtained | Line 196-199: “On the examination report provided by the manufacturer, the epithelial thickness values in the central 7 mm are presented: the upper and lower epithelial thickness in the area of 2 to 7 mm, the minimum and maximum thicknesses as well as the difference between these two thicknesses and topographic epithelium thickness irregularity in the evaluate 7mm area, as reported by the standard deviation (SD) of epithelial thickness measurements (representative examples from the three groups are shown in Figure 2).”  Line 203-205: “The OCT examinations were performed on the same day as the slit lamp examination and the OQAS and the measurements were repeated until a sufficient signal strength index was obtained.” |
| Line 173: a loose trial lens? Did authors check that the trial lens did not impact measurements or introduce additional aberrations? | To avoid a dispersion linked to astigmatism, as indicated by the manufacturer, the patient's astigmatism must be corrected by placing appropriate cylindrical lens on a holder placed in front of the eye. | Line 214-215: “Astigmatism beyond 0.75 diopters was corrected using the appropriate cylindrical lens placed on a holder in front of the eye as advised by the manufacturer.” |
| Line 175: were lighting conditions controlled? Was a standard pupil size used for calculation of the OSI? Is OSI specific to the OQAS instrument? | The image quality of each subject was measured at 4-mm artificial pupil, which was controlled by means of a diaphragm wheel located inside the double-pass system. The diaphragm is conjugated with the pupil plane of the eye and therefore acts as an effective entrance pupil when the natural pupil of the eye is greater than the diaphragm diameter. | Line 213-214: “We acquired double-pass images of every eye at best focus, corrected internally by an optometer, the pupil diameter was set at 4mm” |
| Line 185: were t-tests protected for multiple comparisons? | Thank you for raising this problem, we have redone the statistical analyzes.  The differences between control, DED and EBMD were analyzed using ANOVA followed by post-hoc comparison (RStudio, Boston, MA). | Table 1, 2 and figure 4,5 and 7 |
| Line 192-203: this is a repetition of data already presented in Table 1. To make it easier for the reader, I suggest including statistical comparison of various patient factors between each of the 3 groups in the table. | Thank you for your advice. We have removed the sentences that repeated table 1 and included the statistical comparison in table 1. | Line 192 suppression of “demographic data were presented …”  Line 241-241:” The characteristics of enrolled subjects are reported in table 1.” |
| Line 209-216: this is again a repetition of data already presented in Table 2. Table 2 should include SD values. | Thank you for your advice. | We have included the SD value in table 2 and we have deleted the sentences that repeated table 2. |
| Line 300: the protocol limited analysis of corneal epithelium to central, inferior and superior regions | The irregularity was also included in the protocol. The epithelium thickness irregularity is reported by the standard deviation (SD) of epithelial thickness measurements in the 7mm area. | Line 145 “Central, superior, inferior and minimum, maximum corneal and epithelium thicknesses and the topographic thickness standard deviation were measured using a Fourier-domain OCT system (RTVue; Optovue, Inc., Fremont, CA, USA). OSI features were measured with an OQAS (Optical Quality Analysis System, HD Analyzer, Visiometrics SL, Terrassa, Spain).” |
| Line 326: has the OSI score obtained by the OQAS been compared to subjective measures of quality of life/vision? It has been acknowledged in previous studies that BCVA is not a sensitive measure of changes in visual quality. | It is true that to compare the OSI to a subjective scale of quality of vision would have been very interesting. Unfortunately, we have not obtained these informations in our study population. | Line 607-610: “Herbaut et al. showed that quality of vision measured with the OQAS was correlated with dry eye symptoms and signs (29). It would be interesting to study if, as we think, the OSI index is also correlated with the subjective quality of vision reported by patients with EBMD. In this present study, it should be noted that the OSI measured by the OQAS was more sensitive in detecting this vision impairment than the BCVA.” |
| Line 358: there was also no grading for EBMD. | We add this limitation in the discussion. | Line 665-668:“Another limitation of the study is that we did not grade the severity of patients with EBMD or DED. It would be interesting to study if the irregularity of the epithelium is more important that the dystrophy is severe or that the functional complaints are important.” |
| Line 369: another limitation is that some patients included in the analysis had PCIOL which will | Thank you for you remark, we have added a paragraph to discuss this point. | Line 665-670; “Among the patients included, some had a PCIOL which could bias the analysis of the quality of vision in OSI. The difference in lens status was not statistically significant between the three groups. And in any case, the EBMD group included the largest proportion of PCIOL, thus, the fact that we find a poorer OSI score in this group seems to be linked to the corneal dystrophy rather than the transparency of the lens.” |
| Line 371: How does age affect epithelial thickness? |  | Line 660-662: “according to Kim et al. and Yang et al. the central epithelial thickness remains constant with age while the superior area becomes thinner” |

**Response to reviewer #2**

| Comment | Response | Changes made to the original version |
| --- | --- | --- |
| Could you add a figure of cornea with EBMD with labelling the characteristic signs of finger print lines and microcysts. | Thank you for your advice, we add slit lamp photography of cornea with EBMD | Figure 1: Slit-lamp photography of a cornea with EBMD, fingerprint lines, geographic map-like lines, and epithelial dot are designated by arrows. |
| Would be good idea to mention specific rationale of this research. | We edited the introduction so that the rational of this research appears clearer. | Line 136-138: “Thus, in this study, we aim to investigate the features of the corneal epithelial Fourier-domain OCT thickness map in EBMD patients and to assess the relationship between visual quality and OCT thickness mapping in EMBD compared to DED and normal patients.” |
| Stats: Appropriate sample size calculations (especially effect size) are missing. Did the authors check the normality of the data distribution if yes, then please mention the statically tests used to check the normality.  "A Student t-test was used to compare means between two groups." which groups?. Earlier, authors mentioned three groups , EBMD, DED and normal. | Thank you for raising this problem, we have redone the statistical analyzes.  The differences between control, DED and EBMD were analyzed using ANOVA followed by post-hoc comparisons (RStudio, Boston, MA). The Shapiro-Wilk test was used to evaluate each variable for normality. | Table 1, table 2, figure 4, 5 and 7 |
| OSI is an ordinal - non linear scale so Pearson correlation may not be ideal one to use. | Thank you for your remark, we have redone the correlation analysis using the Spearman method. | Table 3 |
| Diagnostic efficacy of OCT was not your primary or secondary aim. I would suggest to mention the research gap related to diagnostic efficacy of OCT in intro. | Thank you for your advice, we added the mention of the research gap related to diagnostic efficacy of OCT in the introduction | Line 72-73: “ However, there are very few articles in the literature that assess the diagnostic effectiveness of OCT in corneal dystrophies”  Line 105-108: “Therefore, we investigated the features of the corneal epithelial Fourier-domain OCT thickness map in EBMD patients compared to normal subjects and DED patients and we aimed to assess the diagnostic capacity of OCT for this pathology.” |
| Authors needs to justify the reason behind conducting multiple t-test , as this my result in greater type I error. An ANOVA with post-hoc for multiple comparisons would be ideal to control this errors. | Thank you for raising this problem, we have reworked the statistical analyzes.  The differences between control, DED and EBMD were analyzed using ANOVA followed by post-hoc comparisons tests (RStudio, Boston, MA). | Table 1, table 2, figure 4,5 and 7 |
| Correlations: Correlations need to be tabulated. I find these correlations hard to understand. | Thank you for you advice we added a correlation table for a better understanding. | Table 3 |
| I suspect corneal epithelial nerve plexus could be affected in EBMD. So it would be ideal to check the corneal sensitivity in different quadrants and assess its association with other parameters and also could be a part of ROC analysis. | Thank you for raising this very interesting point. The distribution of nerve density with its trophic role for the epithelium may also play a role. We added this point in the discussion. | Line 552: “Furthermore, differences in epithelial nerve density in different corneal regions could also be involved and should be studied. Indeed, Bouheraoua et al. were able to show that decreased subbasal nerve plexus density led to a thinned corneal epithelium after 360-degre laser retinopexy (26).” |
| I suggest a separate ROC analysis between EBMD and DED (with no controls). This will result in better understanding of the diagnostic performance of OCT(Irregularlity) in differentiating both conditions. | Thank you for your advice, we conducted a separate analysis between EBMD and DED and found a good diagnostic power of the irregularity of the epithelium (AUC 0.87). | Line 475-476: “The irregularity of the epithelium was also able to discriminate EBMD and DED subjects (AUC=0,88) (supplementary figure 1).”  Line 573: “The ROC results show the diagnostic performance of the irregularity in EBMD.” |
